# Redefining testosterone reference ranges for adult females

**DOI:** 10.1007/s40618-026-02929-w

**Published:** 2026-06-24

**Authors:** Esther Wainwright, Natalie Getreu, Lauren Crawford, Adrian Timpson, Sofia Vaz, George Adams, Lucinda Lawrie, Timothy Woolley, Lorna Brightmore, Ali Abbara, Helen C. O’Neill

**Affiliations:** 1 Hertility Health, Research and Development, London, W1W 5PF UK; 2 EGA Institute of Women’s Health, University College London, London, WC1E 6HU UK; 3 Department of Genetics, Evolution and Environment, University College London, London, WC1E 6BT UK; 4 Inuvi Diagnostics, Gloucester, GL2 8AX UK; 5 Department of Metabolism, Digestion and Reproduction, Imperial College London, London, UK

**Keywords:** Testosterone, Female, Reference ranges

## Abstract

**Purpose:**

To define an age-continuous reference range for testosterone (T) in females.

**Methods:**

A large-scale retrospective analysis of anonymised serum T collected via capillary sampling from a private healthcare service. 5,323 female individuals (19–59 years) with no history of reproductive conditions, normal BMI and regular cycles were analysed to establish a robust, age-continuous reference range for T, including probability density distributions and specific quantiles. Competing models of log T values as a function of age were statistically assessed, and the best model selected. Additionally, a cohort with self-reported hirsutism was compared.

**Results:**

A weakly skew-Normal distribution describes the best-fit distribution of log T values at any specific age, such that the mean decreases and the variance increases linearly with age. Analysis of the hirsutism cohort showed significantly higher T levels compared to the reference cohort.

**Conclusion:**

Establishing a reliable reference range for female T enables clinicians to differentiate between physiological and pathological states (e.g. hyperandrogenism). Here, we establish a robust, age-continuous reference range for T in a large female population under strict inclusion criteria. The findings underscore the importance of age-specific reference ranges and highlight a substantial decrease in T over adult life. This study provides a valuable tool for clinicians and researchers seeking to compare patient hormone levels to a reliable reference distribution of T levels in women.

**Supplementary Information:**

The online version contains supplementary material available at 10.1007/s40618-026-02929-w.

## Introduction

Testosterone (T) is an androgenic steroid hormone that plays an essential role in female physiology. In women, T is primarily produced in the ovaries and adrenal glands, with peripheral conversion from androgenic precursors such as androstenedione and dehydroepiandrosterone sulfate (DHEAS) also contributing significantly to circulating levels [1]. Once secreted, T circulates in the bloodstream both protein-bound to sex hormone-binding globulin (SHBG) and in unbound (free) forms, with the latter considered biologically active. T exerts effects on libido, mood, bone mineral density, muscle mass, metabolic function, and overall endocrine homeostasis [2–5]. There is a growing interest in T replacement therapies, including creams and gels, which are becoming widely available online and through private companies, particularly in peri- and post-menopausal women, [4, 6–8], however there remains a paucity of data regarding efficacy aside from for sexual symptoms and long-term safety. Further, increasing emphasis has been placed on alternative androgen pathways, particularly 11-oxygenated androgens, which may contribute up to half of total androgen action and show relatively little decline across the menopausal transition [9]. Clinically, T is also part of polyendocrine metabolic ovarian syndrome (PMOS) assessment [10, 11] and other endocrinological diseases including ovarian hyperthecosis [12] and androgen deficiency [13].

T levels fluctuate across the menstrual cycle, declining gradually with age (albeit not dramatically during the menopausal transition), and are modulated by physiological factors such as BMI, stress, and diurnal variation [14–17]. These factors complicate efforts to define what constitutes a ‘normal’ T range in women and make a one-size-fits-all reference range impractical. As a result, establishing a suitable cohort for a reference population requires careful stratification of data after considering the influence of several factors including age, BMI, use of hormonal contraception, pubertal status, menstrual cycle phase, trimester of pregnancy, and menopausal stage.

Typically, reference ranges for analytes are established by collecting many measurements from a reference population and calculating the 95% interval (between the 2.5th and 97.5th percentiles) using either parametric (requiring a normal distribution, often with data transformation) or non-parametric methods [18]. Recently, the International Federation of Clinical Chemistry (IFCC) have emphasised the importance of using ‘decision limits’, i.e. universally agreed upon thresholds, in addition to reference ranges to aid the clinical interpretation of results [19]. However, there has been no national or international consensus of the decision limits to enable diagnosis for specific reproductive health conditions. Further, while thresholds are often chosen with specificity or sensitivity metrics, any single threshold used to create categories is ultimately a chosen point on a continuous scale, and unfairly pushes similar observations that are close to a boundary into different categories. This problem of discontinuous thinking is pervasive in various areas of medical statistics (perhaps most notably the arbitrary significance levels when reporting p-values), but can be overcome when comparing an individual to any reference distribution, by directly reporting the quantile of the reference distribution corresponding to the individual’s observed measurement. This avoids the need to convert the measured analyte value directly into a ‘clinical decision’, and instead retains it as one of several pieces of evidence to be considered together by the clinician in forming a diagnosis. 

Whilst Liquid Chromatography-Tandem Mass Spectrometry (LC–MS/MS) is the recommended gold standard for T measurement, in the UK and Republic of Ireland immunoassay remains the most common, due to cost and complexity [14, 20]. The reference ranges used on these platforms are typically derived from relatively small validation cohorts [12]. For example, the Roche reference range, one of the leading suppliers in the UK, is based on a study of only 149 women, stratified into two broad groups: 20–49 years (n = 89) and ≥ 50 years (n = 71) [21]. This relatively small cohort was only stratified to ensure the participants had normal BMI, and did not use hormonal contraception, but did not exclude other potentially confounding factors such as hirsutism (excess facial/body hair growth). The current reference range provided by Roche is not age-continuous and does not provide continuous quantiles or a probability distribution. Whilst previous studies have aimed to establish reference ranges in immunoassays with larger sample sizes [22, 23], the largest recent study used 95% confidence intervals grouped from less than 500 Iranian women across ‘reproductive age’ [24]. More recent large cohort studies, such as Du et al. (2016; n = 1,590) and Wen et al. (2021; n = 948) have reported reference intervals for female androgens within broader endocrine investigations in Chinese women [25, 26], though these were not specifically designed to define T reference ranges. Further, individual NHS and private laboratories typically rely on reference ranges provided by their specific assay manufacturers or develop local reference intervals through internal validation. As a result, reference ranges vary between trusts and testing sites. This lack of standardisation undermines the comparability of results across clinical settings, potentially leading to inconsistent diagnoses and management decisions for patients tested in different locations.

Therefore, the primary objective of this study is to provide an improved age-specific reference range of total T, using a large cohort of women after excluding for multiple potentially confounding factors. 

## Materials and methods

### Data collection

This study involved the retrospective analysis of data collected from women across England, Scotland, and Wales who accessed a private healthcare service and completed an online health assessment via Hertility Health Ltd between September 2020 and August 2025. The service provides at-home reproductive hormone testing upon completion of an online health assessment (OHA) detailing comprehensive data on demographics, medical history, symptoms, and menstrual cycle. Capillary blood was self-sampled via an at-home finger prick test on day three of their menstrual cycle, after at least an 8-h fasting window. Capillary blood testing has been shown to be comparable to venous sampling [27]. Blood samples were collected in Greiner MiniCollect® CAT Serum Clot Activator Tube (Greiner Bio-One, Kremsmünster, Austria). Serum was separated by centrifugation as per the manufacturer's instructions within three days of sample collection, and the concentrations of T were measured using the Cobas® e801 module (Roche Diagnostics, Basel, Switzerland). This assay has a measuring range of 0.087–52.0 nmol/L. The coefficient of variation (CV) for repeatability and intermediate precision is 1.3–5.7% and 2.0–11.5%, respectively. All assays were performed by Inuvi Diagnostics Ltd. (Gloucestershire, UK), a UKAS-accredited laboratory. This method is traceable to highly purified T by weight via ID‐GC/MS (“Isotope Dilution—Gas Chromatography/Mass Spectrometry”) [28]. 

At the time of participation, all individuals provided consent for their health and personal data to be used in anonymised form for research purposes. Consent was obtained electronically, embedded in the health assessment, with clear information provided about data use, privacy, and confidentiality. As the study involved only retrospective, anonymised data, additional ethical review was not required. This study was approved by the UCL-RFH biobank for sub-study, application, ‘Identifying Hormonal and Cycle Pattern Deviations in a Diverse Female Population from Hertility Health’s Reproductive Dataset’. The biobank is approved under REC reference 21/WA/0388.

### Study population

Our initial dataset comprised 30,453 women aged 18–74 years. After applying exclusion criteria (Table 1) widely used in population-based hormone reference studies to ensure a representative baseline for physiologically typical individuals [5, 29–31], a reference cohort of 5,323 individuals aged 19–59 years was available for statistical analysis. Symptoms of hirsutism were self-defined and a picture scale used to report severity in line with the gold-standard Ferriman-Gallwey assessment [32, 33]. Figure SI1 shows frequency of exclusions and their co-occurrences. Demographic characteristics of the reference and hirsutism cohorts are shown in Table 2.

**Table 1:** Exclusion criteria selected based on known disruptions to T levels and widely used in population-based hormone reference studies [5, 29, 30, 34]

Category	Exclusion criteria
Reproductive conditions (based on previous diagnosis and new diagnosis post-testing)	Polycystic ovarian syndrome (PCOS) Hyper- or hypothyroidism (including subclinical), premature ovarian insufficiency or menopause, Hypothalamic amenorrhea, endometriosis fibroids
Anthropometric Menstrual Factors	BMI > 30 or < 18.5 kg/m^2^ Menstrual cycle length < 21 or > 35 days Period length > 7 or < 2 days
Other factors known to affect T levels	Using hormonal contraception/medication Breastfeeding Symptoms of hirsutism Insulin resistance including type 1 and 2 diabetes

Frequencies and co-occurrences are visualised in Figure SI1

**Table 2:** Demographic information of those included in the final analysis for both the reference cohort (n = 5,323) and the hirsutism cohort (n = 2,338)

Characteristic	Mean ±SD [50% CI] (95% CI) <99% CI> {range}		England and Wales 2021 Census data
	Reference cohort	Hirsutism cohort	
Age (years)	32.3 ± 4.8 [29.3–34.9] (23.8–43.4) <21.1–48.5> {19.1–58.6}	32.4 ± 4.7 [29.4–35.0] (23.8–43.2) <21.0–46.5> {18.9–50.1}	
BMI (kg/m^2)^	23.5 ± 2.8 [21.3–25.5] (19.0–29.4) <18.6–29.8> {18.5–30.0}	24.3 ± 2.9 [22.0–26.6] (19.4–29.6) <18.8–29.9> {18.5–30.0}	
Total Testosterone (nmol/L)	1.1 ± 0.8 [0.7–1.4] (0.3–2.3) <0.1–3.2> {0.1–34.5}	1.3 ± 0.8 [0.8–1.6] (0.3–2.7) <0.2–3.5> {0.1–18.8}	
**Sample size (Percentage)**			
Ethnicity			
White	4456 (83.7%)	1863 (79.7%)	(81.7%)
Black	95 (1.8%)	80 (3.4%)	(4.0%)
Asian	295 (5.5%)	178 (7.6%)	(9.3%)
Mixed	254 (4.8%)	122 (5.2%)	(2.9%)
Other	223 (4.2%)	95 (4.1%)	(2.1%)

BMI = body mass index, SD = standard deviation

### Statistical analysis

We performed three key analyses. Firstly, we graphically evaluated the T values from 5,323 samples to identify general features of the relationship with age. Secondly, we used these general features to propose several plausible age-continuous statistical models for the distribution of log T levels, calculate maximum likelihoods, select the best model, and report the model parameters. This provides a continuous probability distribution (from which preferred quantiles can be calculated) for the expected distribution of log T values for women at any specific age and can be used to assess a patient’s T measurement when no a priori measurement is available for that patient. Thirdly, we analysed the continuous age relationship between T levels and hirsutism to identify elevated T levels in the hirsutism group, which provides support for our decision to exclude these patients from our primary analysis.

**Fig. 1:**
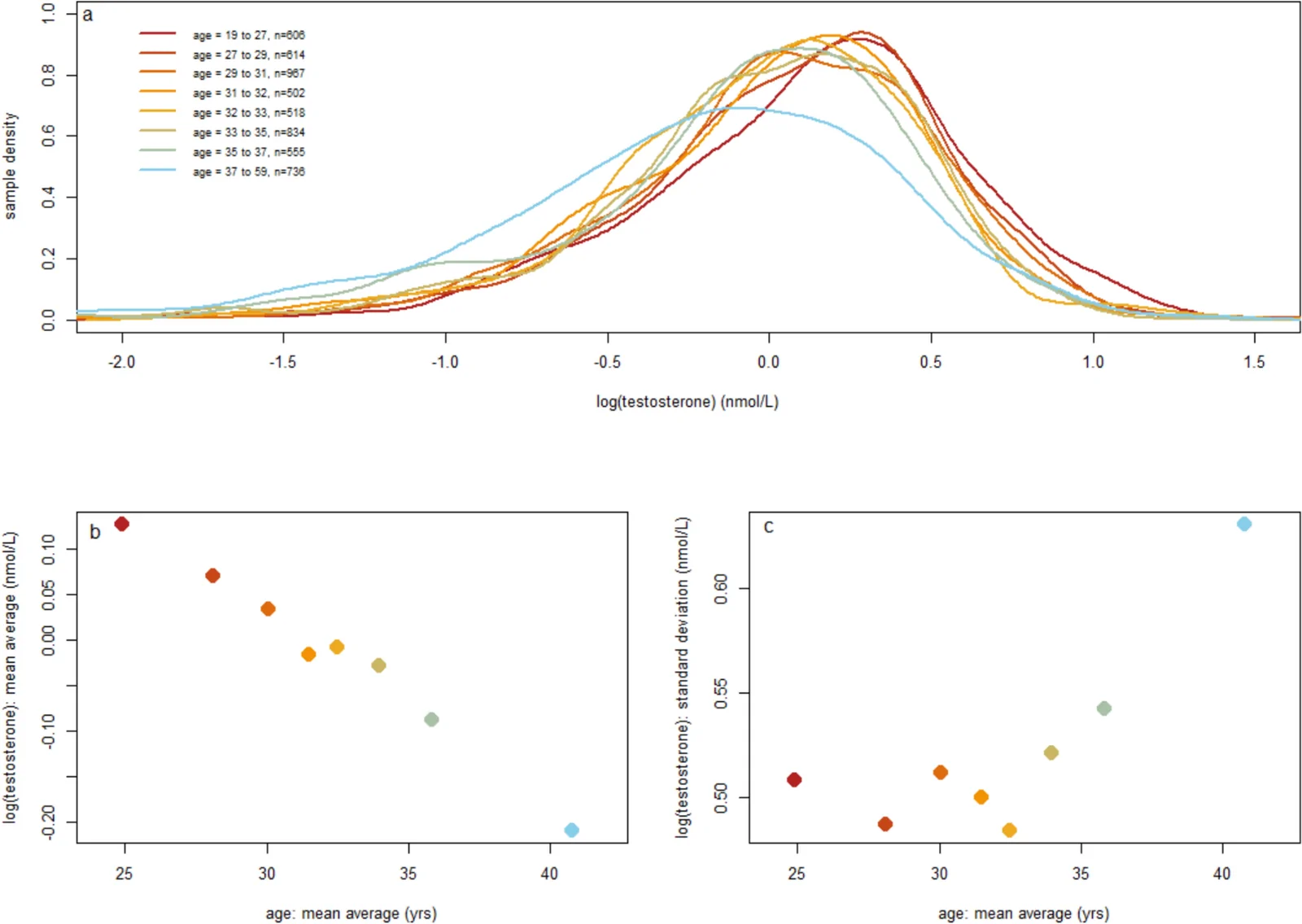
Empirical evaluation. **a** Kernel Density (KD) distributions of log(T) values for 8 age-groups. **b** and **c** mean and SD for each of the age groups. Age brackets were chosen to ensure similar sample sizes in each (12.5% quantiles rounded to the nearest integer year). Together, they illustrate a continuous trend of decreasing mean T levels with increasing age and increasing SD in the population with increasing age. KD bandwidth selected automatically using the default nrd0 algorithm in the R function *density* (stats package) [35]

Analysis 1: To graphically evaluate our dataset of 5,323 measurements from the primary reference cohort, we grouped the data into eight discrete age classes. Age bounds for each discrete age group were systematically chosen as the eight quantiles (0%, 12.5%, 25%, 37.5%, 50%, 62.5%, 75%, 87.5% and 100%) of the overall age distribution, rounded to the nearest year, thus ensuring a large sample size in each (n = 502 to n = 967). Within each group, we used Kernel Density Estimation to construct an empirical distribution of log T values. Further, within each group, we calculated both the mean and standard deviation (SD) of log T values and plotted them against mean age (Fig. 1). Kernel density bandwidth was selected automatically using Silverman’s rule of thumb [33], the default heuristic algorithm in the R function *density* (stats package) [35].

Analysis 2: Initial data representation from analysis 1 revealed several features that assisted us in forming six plausible models of log T levels of varying complexity, predicting how log T values vary as a function of age. By considering an age-continuous model of log T values, we leveraged the large sample size across the full age range of our data (19–59 years). For each model, we performed a maximum likelihood search of parameter space, and performed model comparison using the Bayesian Information Criterion (BIC) [36], which optimises the balance between goodness of fit (likelihoods) whilst penalising for too much model complexity (number of parameters). Additionally, we calculated a summary table of quantiles (at each year of age) from the best model.

Analysis 3: We utilised the best age-continuous model selected from analysis 2, and repeated the parameter search using 2,338 T values from a cohort of patients who self-reported hirsutism. The cohort met all other exclusion criteria used in analysis 2. We then compared the modelled distribution of log T values between the reference and hirsutism cohorts.

## Results

The empirical distributions from analysis 1 (Fig. 1) suggested several features that assisted us in forming plausible age-continuous models of log T levels. Firstly, they suggested a Normal or slightly skew-Normal distribution may be a sensible model component (Fig. 1a), at any age. Secondly, we observed that mean log T levels decrease as age increases (Fig. 1b), and that this relationship is well represented with a simple monotonic relationship. Thirdly, we observed an increase in log T SD with age (Fig. 1c), which is also well represented with a monotonic linear relationship. Therefore, these initial observations formed the basis of our six models in analysis 2.

**Fig. 2:**
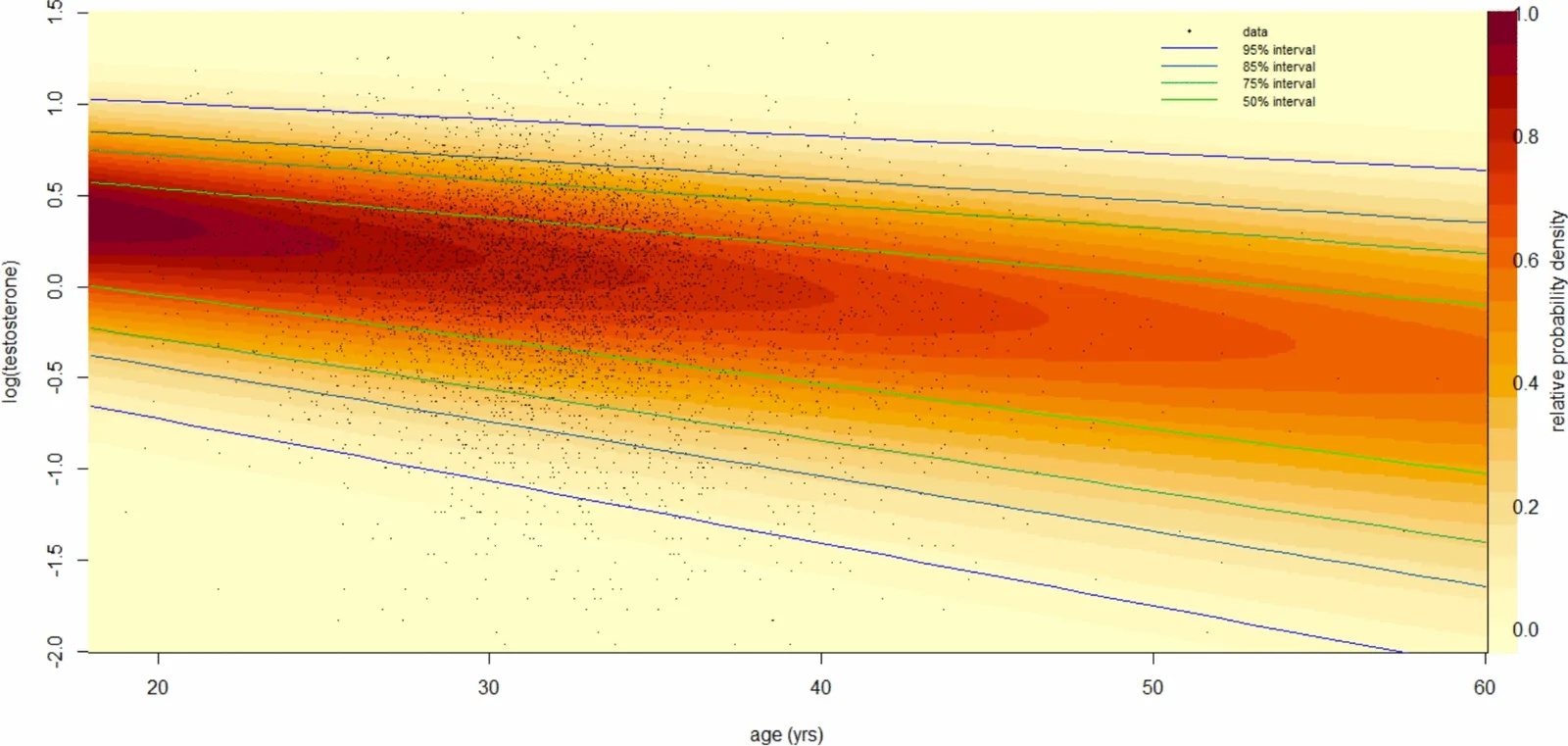
Probability density and confidence intervals of age-continuous best fitting model 4 overlayed with raw data (N = 5323), log_e_(T) ~ SN(ξ, ω, α) where ξ = -0.014233(age) + 0.948679, ω = 0.008922(age) + 0.440994 and α = -1.833742

Results of the six age-continuous models from analysis 2, including the Maximum Likelihood Estimates (MLE) of their corresponding parameter values, are presented in Table 3. Model 4 is selected as the best (lowest BIC), in which the expected log T value decreases linearly with age, and the variance in log T increases linearly with age. Figure 2 illustrates the probability density of this best-fitting model, along with the 50%, 75%, 85% and 95% intervals. The total T values for several quantiles are also summarised in Table 4. Model 4 estimates this change in log T at -0.0142 per year, equivalent to a 1.4% annual decrease in raw total T values.

**Table 3:** Model summary of six plausible age-continuous models of log T values, including Maximum Likelihood Estimates (MLE) of parameter values, the overall Log Likelihoods, the number of parameters (N pars), and the Bayesian Information Criterion (BIC)

Model	Description	Formal description	MLE	Log likelihood	N pars	BIC
Model 1	Gaussian distribution with fixed σ, but μ is a linear function of age	log(T) ~ N(μ, σ^2^) where: μ = m(age) + c	m = -0.020837 c = 0.656490 σ = 0.526739	-4140.697	3	8307.133
Model 2	Gaussian distribution where μ is a linear function of age, and σ is a linear function of age	log(T) ~ N(μ, σ^2^) where: μ = m_1_(age) + c_1_ σ = m_2_(age) + c_2_	m_1_ = -0.020338 m_2_ = 0.006392 c_1_ = 0.640456 c_2_ = 0.319069	-4120.087	4	8274.493
Model 3	Skew gaussian distribution with fixed ω, fixed α, but ξ is a linear function of age	log(T) ~ SN(ξ, ω, α) where: ξ = m(age) + c	m = -0.018280 c = 1.079791 ω = 0.730435 α = -1.830159	-4017.435	4	8069.189
Model 4	**Skew gaussian distribution where ξ is a linear function of age, and ω is a linear function of age, and α is fixed**	**log(T) ~ SN(ξ, ω, α)** **where:** **ξ = m**_**1**_**(age) + c**_**1**_ **ω = m**_**2**_**(age) + c**_**2**_	**m**_**1**_** = -0.014233** **m**_**2**_** = 0.008922** **c**_**1**_** = 0.948679** **c**_**2**_** = 0.440994** **α = -1.833742**	**-3996.898**	**5**	**8036.695**
Model 5	Skew gaussian distribution where ξ is a second degree polynomial function of age, and ω is a linear function of age, and α is fixed	log(T) ~ SN(ξ, ω, α) where: ξ = a(age^2^) + b(age) + c_1_ ω = m(age) + c_2_	a = 0.000138 b = -0.005125 c_1_ = 0.800782 m = 0.008910 c_2_ = 0.440877 α = -1.829342	-3996.614	6	8044.707
Model 6	Skew gaussian distribution where ξ is a third degree polynomial function of age, and ω is a linear function of age, and α is fixed	log(T) ~ SN(ξ, ω, α) where: ξ = a(age^3^) + b(age^2^) + c_1_(age) + d ω = m(age) + c_2_	a = -0.000001 b = 0.000023 c_1_ = -0.008992 d = 0.843099 m = 0.008912 c_2_ = 0.440931 α = -1.829511	-3996.613	7	8053.284

Values found using a differential evolution optimisation algorithm (*JDEoptim* function from R package DEoptimR) [37]. Model selection favours Model 4 (lowest BIC), such that both the expected T values and the variance are linear functions of age

**Table 4:** Total T values (nmol/L) for 95%, 85%, 75%, and 50% confidence intervals (CI), at 60 discrete ages

Age	Q 0.025	Q 0.075	Q 0.125	Q 0.25	Q 0.75	Q 0.875	Q 0.925	Q 0.975
18	0.519	0.685	0.794	1.002	1.771	2.111	2.339	2.796
19	0.502	0.665	0.772	0.978	1.743	2.083	2.312	2.770
20	0.485	0.645	0.751	0.954	1.715	2.056	2.284	2.745
21	0.468	0.626	0.731	0.931	1.688	2.028	2.257	2.720
22	0.453	0.607	0.710	0.909	1.661	2.001	2.231	2.695
23	0.437	0.589	0.691	0.887	1.635	1.974	2.204	2.670
24	0.423	0.572	0.672	0.865	1.609	1.948	2.178	2.645
25	0.408	0.555	0.653	0.844	1.583	1.922	2.152	2.621
26	0.395	0.538	0.635	0.824	1.558	1.896	2.127	2.597
27	0.381	0.522	0.618	0.804	1.533	1.871	2.102	2.573
28	0.369	0.507	0.601	0.785	1.509	1.846	2.077	2.549
29	0.356	0.492	0.584	0.766	1.485	1.822	2.052	2.526
30	0.344	0.477	0.568	0.747	1.461	1.797	2.028	2.502
31	0.333	0.463	0.553	0.729	1.438	1.773	2.004	2.479
32	0.321	0.449	0.537	0.711	1.415	1.750	1.980	2.456
33	0.311	0.436	0.523	0.694	1.393	1.726	1.957	2.434
34	0.300	0.423	0.508	0.677	1.371	1.703	1.934	2.411
35	0.290	0.410	0.494	0.661	1.349	1.681	1.911	2.389
36	0.280	0.398	0.481	0.645	1.327	1.658	1.888	2.367
37	0.271	0.386	0.467	0.630	1.306	1.636	1.866	2.345
38	0.262	0.375	0.454	0.614	1.286	1.614	1.844	2.324
39	0.253	0.364	0.442	0.599	1.265	1.593	1.822	2.302
40	0.244	0.353	0.430	0.585	1.245	1.572	1.801	2.281
41	0.236	0.343	0.418	0.571	1.225	1.551	1.779	2.260
42	0.228	0.332	0.406	0.557	1.206	1.530	1.758	2.239
43	0.221	0.323	0.395	0.544	1.187	1.510	1.737	2.219
44	0.213	0.313	0.384	0.530	1.168	1.490	1.717	2.198
45	0.206	0.304	0.374	0.518	1.149	1.470	1.697	2.178
46	0.199	0.295	0.364	0.505	1.131	1.450	1.676	2.158
47	0.192	0.286	0.354	0.493	1.113	1.431	1.657	2.138
48	0.186	0.277	0.344	0.481	1.095	1.412	1.637	2.118
49	0.180	0.269	0.334	0.469	1.078	1.393	1.618	2.099
50	0.174	0.261	0.325	0.458	1.061	1.374	1.599	2.080
51	0.168	0.253	0.316	0.447	1.044	1.356	1.580	2.060
52	0.162	0.246	0.307	0.436	1.027	1.338	1.561	2.041
53	0.157	0.239	0.299	0.426	1.011	1.320	1.543	2.023
54	0.151	0.232	0.291	0.415	0.995	1.302	1.524	2.004
55	0.146	0.225	0.283	0.405	0.979	1.285	1.506	1.986
56	0.141	0.218	0.275	0.395	0.963	1.268	1.488	1.967
57	0.137	0.212	0.267	0.386	0.948	1.251	1.471	1.949
58	0.132	0.205	0.260	0.377	0.933	1.234	1.453	1.931
59	0.128	0.199	0.253	0.367	0.918	1.218	1.436	1.913
60	0.123	0.193	0.246	0.359	0.904	1.202	1.419	1.896

Extracted from the age-continuous model, providing the CI at the start of each age, not throughout the year of each age

**Fig. 3:**
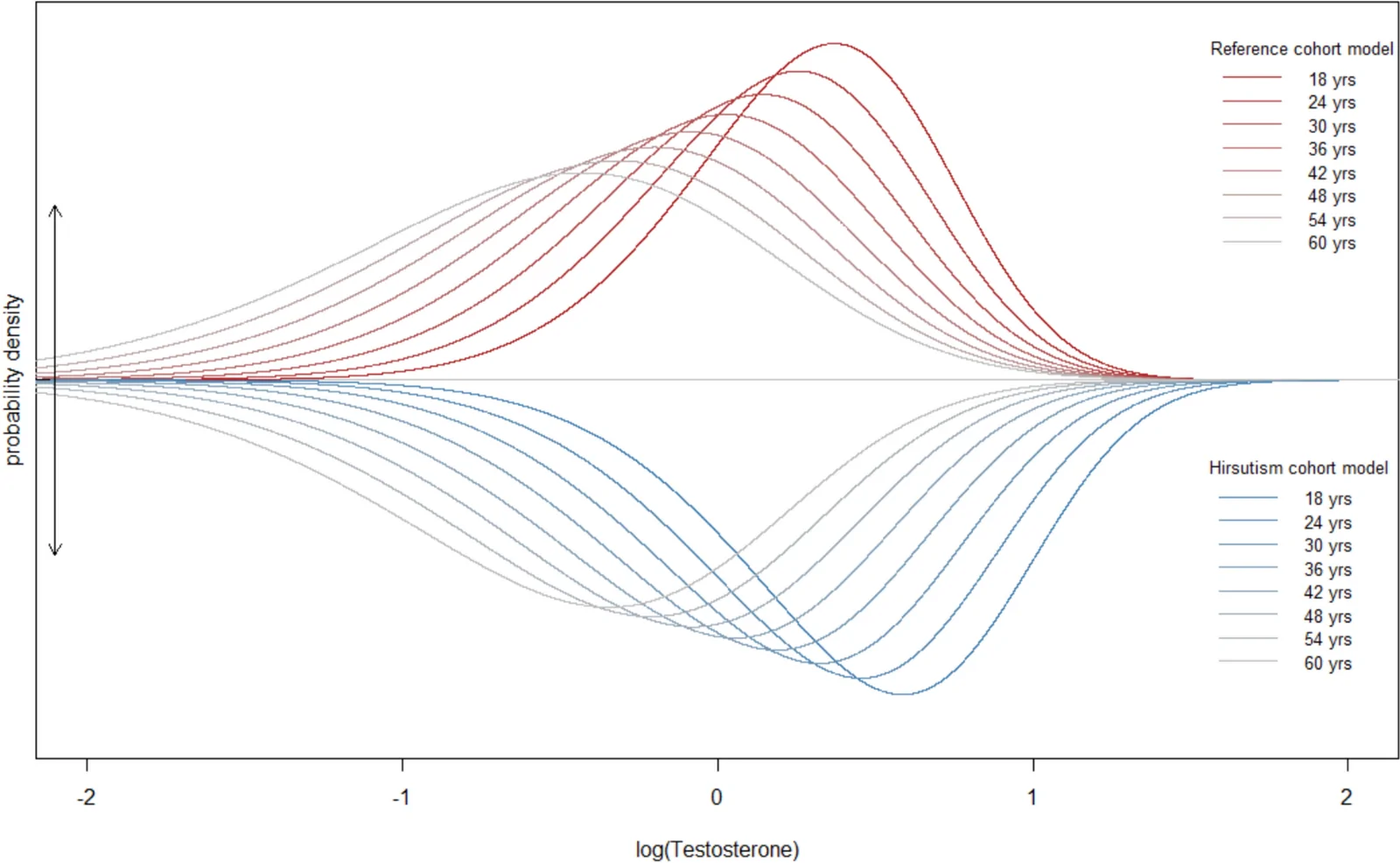
Probability Density Distributions for Log T levels derived from model 4, stratified by age, and grouped by individuals with (n = 2,338) or without (n = 5,323) hirsutism. T levels in both groups decrease with age. Significant differences exist between both groups at age 18 years, but these differences decay with age

Results from analysis 3 comparing the best fit model for a cohort of women with hirsutism against the best fit model for the reference cohort indicate the former have, on average, higher log T levels (Fig. 3). The difference is most pronounced at age 18 years, where the mode of log T values for the hirsutism group is 0.5868, 0.2176 greater than the normal cohort (0.3692). With increasing age, the differences between the two cohorts shrink, such that by age 60, the mode of the hirsutism cohort is only 0.0849 greater. Nevertheless, there remains significant overlap between the two groups at all ages.

## Discussion

To our knowledge, this study represents the largest analysis to-date of female total T levels, defining an age-continuous reference range across 5,323 rigorously screened women aged 19–59 years. Using advanced statistical modelling, we demonstrated that a weakly skew-Normal distribution best describes the relationship between log-transformed T and age, with mean levels declining by approximately 1.4% per year and variance increasing linearly with age. These findings confirm a progressive decline in T throughout adult life and underscore the necessity of age-specific reference intervals rather than static categorical thresholds. By excluding individuals with self-reported hirsutism—who exhibited significantly higher T values, particularly in younger age groups—this study provides a clinically robust benchmark that describes the full spectrum of age-specific androgen levels in women. The resulting model enables clinicians to interpret results on a probabilistic rather than binary basis, enhancing diagnostic precision and personalisation for hyperandrogenic conditions such as PCOS, and improving the comparability of hormone testing across laboratories.

Analysis 2 provides a continuous age-dependent model of healthy log T values for females aged 18–59 yrs. This is a key finding, and the model formula can be used to input a patient’s age to calculate the quantile of the reference distribution corresponding to the patient’s T value. Alternatively, Table 4 provides an easily interpretable summary of age-specific quantiles, allowing rapid comparison of individual T values against population norms for clinicians.

Our age-continuous model also provides insight into the relationship between age and total T values in females, with an estimated linear 1.4% annual decline. Previous work has also identified a progressive decline in T levels in women [38–41]. However, others have suggested a more complex relationship between age and T values, particularly in perimenopausal women [30, 42]. Whilst total T may show a variable decline dependent on age, longitudinal studies have found free T to steadily decline up until menopause [38]. This further underpins the necessity for age appropriate references of T across a woman's reproductive lifespan. Further, we provide evidence of increased variation within total T in women as they age. In principle, we should expect differences between individuals in the rate at which T decreases with age. We should therefore expect the population variance to increase with age, merely as a statistical consequence of increasing divergence with age. Indeed, this is exactly what we observe (Figs. 1, 2). As such, the results from analysis 2 provide us a reference range to assess how typical a single observation from one patient is, when no prior observations are available. To provide a more accurate estimate of age-related decline in total T in women across the reproductive life span and individual long-term variation, further longitudinal studies are needed. Future research should particularly prioritise the inclusion of younger women, as most existing longitudinal datasets have predominantly focused on older cohorts [38, 42], limiting our understanding of early-life trajectories in androgen decline.

While the 99% confidence interval of our reference cohort spans 0.1 to 3.2 nmol/L (Table 2), five individuals (<0.1% of the overall cohort) presented with extreme testosterone values exceeding the 10 nmol/L threshold historically utilised by the International Olympic Committee for female athletic competition eligibility [43]. Specifically, these values ranged from 13.0 to 34.5 nmol/L (13.0, 13.9, 20.9, 21.6, and 34.5 nmol/L). None of these individuals reported a history of reproductive conditions, and their corresponding gonadotropin profiles showed no signs of external hormone suppression, with LH ranging from 5.0 to 16.7 IU/L and FSH ranging from 7.2 to 15.8 IU/L. When log-transformed, these data points align with the heavy tail of the distribution rather than manifesting as arbitrary statistical outliers. This phenomenon is consistent with broader epidemiological data; for instance, in the Sönksen et al. (2018) study of elite athletes [44], 11 of 231 dietary and anthropometrically baseline-typical females (5.2%) exhibited testosterone levels above 10 nmol/L, with five ranging between 21.2 and 31.9 nmol/L. Potential explanations for these elevated profiles in our cohort include unverified exogenous exposure, rare underlying pathologies such as an androgen-secreting neoplasm, or assay-specific anomalies. Because these rare cases do not meaningfully disrupt the overall model parameters due to our expansive sample size, they were retained to maintain statistical integrity, though further clinical work is required to map the exact etiology of such extreme variations in the general population.

Realistically, not all laboratories will be able to calculate their own reference ranges, leading to the over-utilisation of historic reference ranges from populations which may not be truly representative or sufficient for clinical interpretation [45]. A recent review of laboratory practice in the US identified that only 10% of laboratories that assay reproductive hormones have conducted their own reference range studies, of which reference populations have ranged between 20 and 200 participants [46]. The IFCC have suggested ‘indirect’ approaches of forming a reference population, whereby all measurements taken from one or more laboratories are pooled and mined in order to obtain a large, heterogeneous reference population [47]. Unfortunately, this approach does not allow the exclusion of participants with a known health issue, meaning its utility for reproductive health screening is limited. Our study has pooled an age diverse sample from across the UK, with detailed health information, allowing for exclusion of conditions associated with elevated T levels.

Analysis 3 identified that individuals who self-reported hirsutism had consistently higher T levels than the reference cohort. This was particularly prominent in younger women—using the best-fitting age-continuous model from analysis 2 (Fig. 3), we found that the mode of log-transformed T values at age 18 was 0.5868 (raw T = 1.80) in the hirsutism cohort, compared to 0.3692 (raw T = 1.45) in the reference cohort, a difference of 0.2176 or 59% greater in the hirsutism cohort (24% greater in raw T). Although this difference narrowed with increasing age, it persisted across the reproductive lifespan, supporting a biological correlation between this self-reported phenotype and hyperandrogenism. These findings validate our decision to exclude individuals with self-reported excess hair from the reference cohort. Hirsutism is a well-established clinical indicator of androgen excess linked to PCOS in women [10]. Self-reported hirsutism is frequently used as a proxy for suspected hyperandrogenism and has been shown to correlate with elevated biochemical markers of androgen excess [48]. Including individuals with suspected hyperandrogenism in the construction of normative reference ranges risks skewing the upper end of the distribution, thereby reducing the sensitivity of these ranges to detect true abnormalities in clinical settings.

### Strengths

This study represents the largest of its kind, with 5,323 women, well beyond the 120 considered the minimum [49] and the 149 women who previously provided samples for the Roche reference ranges [21]. Furthermore, the in-depth medical and reproductive history collected allowed for stringent exclusion criteria, well beyond what is collected in routine pathology data. This stringent exclusion process was further demonstrated through analysis 3, where reporting of excess hair growth correlated with greater T values, particularly at younger ages. All samples were obtained through the same procedure on day three of the cycle after at least an 8-h fast, allowing for accurate comparisons. The robust statistical modelling enabled age-specific quantiles and average rates of decline across a year, far superior to the 31-year age range established by the manufacturer [21]. The combined advantage of significantly larger sample size, highly standardised collection protocols, stringent exclusion criteria, and robust age-specific statistical modelling renders the resulting reference intervals far more accurate and clinically relevant than those previously derived from limited populations and broad age ranges. As this study utilised immunoassay data consistent with routine NHS and clinical practice in the UK and Ireland, findings are directly applicable to the clinical settings in which the majority of testosterone measurements and subsequent management decisions are made.

### Limitations

We acknowledge that this study has some limitations that may affect the interpretation of the results. Samples were collected remotely, resulting in a 24–72 h lag between collection and assay, however serum stability of a least 3 days has been previously verified [50, 51]. The sample is a self-selected convenience sample of test purchasers, which likely results in a dual bias: overrepresenting individuals of higher socioeconomic status and those who are symptomatic or experiencing fertility difficulties. Given the strict exclusion criteria applied, this cohort likely represents a well-screened population actively engaged in reproductive health monitoring. Consequently, the findings may not be generalisable to the broader population. The current exclusion criteria focused on self-reported hirsutism and PCOS diagnosis. However, acne and female pattern hair loss are also key clinical features of hyperandrogenism. The potential inclusion of individuals with T-related acne or hair loss, but without hirsutism or a PCOS diagnosis, represents a limitation, as this may slightly inflate the upper quantiles. We acknowledge that insulin resistance is a common metabolic factor that can influence T [52]. Whilst we did not have direct biochemical measurements of insulin sensitivity for this cohort, we excluded participants with a history of Type 1 or 2 diabetes. The number of individuals reporting this condition was negligible, and as the final reference cohort was restricted to those with a normal BMI and regular menstrual cycles, the impact of undiagnosed insulin resistance is likely minimal.

Further, self-reported data may lead to erroneous reporting of medical history. The reference cohort was predominantly white (white = 83.7%, Asian = 5.5%, Black = 1.8% Mixed = 4.8%, Other = 4.2%). Future studies should expand this model into more diverse populations.

## Conclusion

This large-scale analysis provides a valuable tool for interpreting female serum T levels. Through robust statistical modelling, we establish age-specific quantiles for women aged 18–60 years, offering a continuous reference framework for total T measurement rather than relying on discontinuous categorical ranges. Our quantitative validation of hirsutism as a key exclusion criterion ensures a rigorously defined, representative reference cohort drawn from across the UK. Collectively, these findings offer a clear point of reference for clinicians and researchers, particularly those involved in reproductive health assessment and screening. Expanding this model through integration with additional laboratory datasets will strengthen its applicability across more diverse populations and address current limitations. Future research should focus on modelling the age-related decline of T through longitudinal testing of an individual both annually and sub-annually to provide short-term fluctuations in T levels, and longer-term patterns of hormonal change across the reproductive lifespan.

## Supplementary Information

Below is the link to the electronic supplementary material.

Supplementary file 1.

## Data Availability

The datasets analysed during the current study are not publicly available because they contain sensitive health information collected through Hertility Health Ltd. Anonymised aggregate data may be available from the corresponding author on reasonable request, subject to appropriate data governance approvals.

## References

[1] Walters KA, Handelsman DJ (2018) Role of androgens in the ovary. Mol Cell Endocrinol 465:36–47. 10.1016/j.mce.2017.06.026 28687450 10.1016/j.mce.2017.06.026

[2] Shifren JL, Braunstein GD, Simon JA, Casson PR, Buster JE, Redmond GP et al (2000) Transdermal testosterone treatment in women with impaired sexual function after Oophorectomy. N Engl J Med 343:682–688. 10.1056/NEJM200009073431002 10974131 10.1056/NEJM200009073431002

[3] Miller KK, Biller BMK, Beauregard C, Lipman JG, Jones J, Schoenfeld D et al (2006) Effects of testosterone replacement in androgen-deficient women with hypopituitarism: a randomized, double-blind. Placebo-Controlled Study J Clin Endocrinol Metab 91:1683–1690. 10.1210/jc.2005-2596 16478814 10.1210/jc.2005-2596

[4] Sheffield-Moore M, Paddon-Jones D, Casperson SL, Gilkison C, Volpi E, Wolf SE et al (2006) androgen therapy induces muscle protein anabolism in older women. J Clin Endocrinol Metab 91:3844–3849. 10.1210/jc.2006-0588 16895962 10.1210/jc.2006-0588

[5] Davis SR, Wahlin-Jacobsen S (2015) Testosterone in women—the clinical significance. Lancet Diabetes Endocrinol 3:980–992. 10.1016/S2213-8587(15)00284-3 26358173 10.1016/S2213-8587(15)00284-3

[6] Parish SJ, Simon JA, Davis SR, Giraldi A, Goldstein I, Goldstein SW et al (2021) International society for the study of women’s sexual health clinical practice guideline for the use of systemic testosterone for hypoactive sexual desire disorder in women. J Sex Med 18:849–867. 10.1016/j.jsxm.2020.10.009 33814355 10.1016/j.jsxm.2020.10.009

[7] Scott A, Newson L (2020) Should we be prescribing testosterone to perimenopausal and menopausal women? A guide to prescribing testosterone for women in primary care. Br J Gen Pract 70:203–204. 10.3399/bjgp20X709265 32217602 10.3399/bjgp20X709265 PMC7098532

[8] Duralde ER, Sobel TH, Manson JE (2023) Management of perimenopausal and menopausal symptoms. BMJ 382:e072612. 10.1136/bmj-2022-072612 37553173 10.1136/bmj-2022-072612

[9] Schiffer L, Kempegowda P, Sitch AJ, Adaway JE, Shaheen F, Ebbehoj A et al (2023) Classic and 11-oxygenated androgens in serum and saliva across adulthood: a cross-sectional study analyzing the impact of age, body mass index, and diurnal and menstrual cycle variation. Eur J Endocrinol 188:86–100. 10.1093/ejendo/lvac017 10.1093/ejendo/lvac017 36651154

[10] Teede HJ, Tay CT, Laven JJE, Dokras A, Moran LJ, Piltonen TT et al (2023) Recommendations from the 2023 international evidence-based guideline for the assessment and management of polycystic ovary syndrome. J Clin Endocrinol Metab 108:2447–2469. 10.1210/clinem/dgad463 37580314 10.1210/clinem/dgad463 PMC10505534

[11] Bizuneh AD, Joham AE, Teede H, Mousa A, Earnest A, Hawley JM et al (2025) Evaluating the diagnostic accuracy of androgen measurement in polycystic ovary syndrome: a systematic review and diagnostic meta-analysis to inform evidence-based guidelines. Hum Reprod Update 31:48–63. 10.1093/humupd/dmae028 39305127 10.1093/humupd/dmae028 PMC11696697

[12] Elhassan YS, Hawley JM, Cussen L, Abbara A, Clarke SA, Kempegowda P et al (2025) Society for endocrinology clinical practice guideline for the evaluation of androgen excess in women. Clin Endocrinol (Oxf) 103:540–566. 10.1111/cen.15265 40364581 10.1111/cen.15265 PMC12413683

[13] Mazer NA (2002) Testosterone deficiency in women: etiologies, diagnosis, and emerging treatments. Int J Fertil Womens Med 47:77–86 11991434

[14] Kanakis GA, Tsametis CP, Goulis DG (2019) Measuring testosterone in women and men. Maturitas 125:41–44. 10.1016/j.maturitas.2019.04.203 31133215 10.1016/j.maturitas.2019.04.203

[15] Atukorala KR, Silva W, Amarasiri L, Ferdinando D (2022) Changes in serum testosterone during the menstrual cycle – an integrative systematic review of published literature. Gynecol Reprod Endocrinol Metab 3:9–20. 10.53260/grem.223012

[16] Algburi HD, Alhamza A, Mansour AA (2023) Diurnal variation of serum total testosterone in women: a single-center study from basrah. Cureus. [cited 2025 Dec 15]. 10.7759/cureus.47677 10.7759/cureus.47677 PMC10673631 38021833

[17] Flores-Ramos M, Martínez-Mota L, Silvestri-Tommasoni R, Jiménez Domínguez G, Linares Silva D (2024) Free testosterone is associated with perceived stress in women. Personalized Med Psychiatry 45–46:100129. 10.1016/j.pmip.2024.100129

[18] Solberg HE (1993) A guide to IFCC recommendations on reference values. J Int Fed Clin Chem 5:162–165 10146392

[19] Ozarda Y, Sikaris K, Streichert T, Macri J (2018) Distinguishing reference intervals and clinical decision limits – a review by the IFCC Committee on Reference Intervals and Decision Limits. Crit Rev Clin Lab Sci 55:420–431. 10.1080/10408363.2018.1482256 30047297 10.1080/10408363.2018.1482256

[20] Livingston M, Downie P, Hackett G, Marrington R, Heald A, Ramachandran S (2020) An audit of the measurement and reporting of male testosterone levels in UK clinical biochemistry laboratories. Int J Clin Pract. [cited 2025 Dec 15]:74. 10.1111/ijcp.13607 10.1111/ijcp.13607 32649008

[21] Roche Diagnostics (2020) Elecsys Testosterone II. Roche Diagnostics GmbH, Sandhofer Strasse 116, D-68305 Mannheim. https://elabdoc-prod.roche.com/eLD/api/downloads/206f25cb-69dd-ea11-fe90-005056a772fd?countryIsoCode=XG

[22] Ayala C, Steinberger E, Smith KD, Rodriguez-Rigau LJ, Petak SM (1999) Serum testosterone levels and reference ranges in reproductive-age women. Endocr Pract 5:322–329. 10.4158/EP.5.6.322 15251653 10.4158/EP.5.6.322

[23] Braunstein GD, Reitz RE, Buch A, Schnell D, Caulfield MP (2011) Testosterone reference ranges in normally cycling healthy premenopausal women. J Sex Med 8:2924–2934. 10.1111/j.1743-6109.2011.02380.x 21771278 10.1111/j.1743-6109.2011.02380.x

[24] Meamar R, Feizi A, Aminorroaya A, Amini M, Iraj B, Heidarpour M (2023) Reference range of testosterone and dehydroepiandrosterone sulfate levels in women during reproductive age in the Iranian population. J Res Med Sci. [cited 2026 Jan 5]:28. 10.4103/jrms.jrms_100_22 10.4103/jrms.jrms_100_22 PMC10729679 38116488

[25] Du X, Ding T, Zhang H, Zhang C, Ma W, Zhong Y et al (2016) Age-specific normal reference range for serum Anti-Müllerian hormone in healthy Chinese Han women: a nationwide population-based study. Reprod Sci 23:1019–1027. 10.1177/1933719115625843 26763552 10.1177/1933719115625843

[26] Wen J, Huang K, Du X, Zhang H, Ding T, Zhang C et al (2021) Can Inhibin B reflect ovarian reserve of healthy reproductive age women effectively? Front Endocrinol 12:626534. 10.3389/fendo.2021.626534 10.3389/fendo.2021.626534 PMC8081350 33935966

[27] Vasavan T, Timpson A, Woolley T, O’Neill HC, Getreu N (2025) Systematic reduction in estradiol and testosterone measurements due to serum separator gel in blood collection tubes: implications for at-home fertility testing. J Appl Lab Med. 10.1093/jalm/jfaf040 40314381 10.1093/jalm/jfaf040

[28] Thienpont LM, De Brabandere VI, Stockl D, De Leenheer AP (1994) Use of cyclodextrins for prepurification of progesterone and testosterone from human serum prior to determination with isotope dilution gas chromatography/mass spectrometry. Anal Chem 66:4116–4119. 10.1021/ac00094a041 7810905 10.1021/ac00094a041

[29] Mazza E, Troiano E, Ferro Y, Lisso F, Tosi M, Turco E et al (2024) Obesity, dietary patterns, and hormonal balance modulation: gender-specific impacts. Nutrients 16:1629. 10.3390/nu16111629 38892561 10.3390/nu16111629 PMC11174431

[30] Sowers M, Beebe JL, McConnell D, Randolph J, Jannausch M (2001) Testosterone concentrations in women aged 25–50 years: associations with lifestyle, body composition, and ovarian status. Am J Epidemiol 153:256–264. 10.1093/aje/153.3.256 11157413 10.1093/aje/153.3.256

[31] Ferriman D, Gallwey JD (1961) Clinical assessment of body hair growth in women. J Clin Endocrinol Metab 21:1440–1447. 10.1210/jcem-21-11-1440 13892577 10.1210/jcem-21-11-1440

[32] NICE (2024) Hirsutism: How do I know my patient has it?. NICE. https://cks.nice.org.uk/topics/hirsutism/diagnosis/diagnosis/. Accessed 11 Jan 2026

[33] Silverman BW (2018) Density estimation for statistics and data analysis, First edition. CRC Press, Boca Raton, FL

[34] Van Anders SM, Watson NV (2006) Menstrual cycle irregularities are associated with testosterone levels in healthy premenopausal women. Am J Hum Biol 18:841–844. 10.1002/ajhb.20555 17039468 10.1002/ajhb.20555

[35] R Core Team. R: A language and environment for statistical computing. Vienna, Austria: R Foundation for Statistical Computing; https://www.r-project.org/

[36] Schwarz G (1978) Estimating the dimension of a model. Ann Stat. [cited 2026 Jan 5]:6. 10.1214/aos/1176344136

[37] Conceicao ELT (2014) DEoptimR: differential evolution optimization in pure R. [cited 2026 Jan 5]:1.1–4. 10.32614/CRAN.package.DEoptimR

[38] Fabbri E, An Y, Gonzalez-Freire M, Zoli M, Maggio M, Studenski SA et al (2016) Bioavailable testosterone linearly declines over a wide age spectrum in men and women from the Baltimore Longitudinal Study of Aging. J Gerontol A Biol Sci Med Sci 71:1202–1209. 10.1093/gerona/glw021 26921861 10.1093/gerona/glw021 PMC4978359

[39] Zumoff B, Strain GW, Miller LK, Rosner W (1995) Twenty-four-hour mean plasma testosterone concentration declines with age in normal premenopausal women. J Clin Endocrinol Metab 80:1429–1430. 10.1210/jcem.80.4.7714119 7714119 10.1210/jcem.80.4.7714119

[40] Spencer JB, Klein M, Kumar A, Azziz R (2007) The age-associated decline of androgens in reproductive age and menopausal Black and White women. J Clin Endocrinol Metab 92:4730–4733. 10.1210/jc.2006-2365 17895325 10.1210/jc.2006-2365

[41] Haring R, Hannemann A, John U, Radke D, Nauck M, Wallaschofski H et al (2012) Age-specific reference ranges for serum testosterone and androstenedione concentrations in women measured by liquid chromatography-tandem mass spectrometry. J Clin Endocrinol Metab 97:408–415. 10.1210/jc.2011-2134 22162468 10.1210/jc.2011-2134

[42] Burger HG, Dudley EC, Cui J, Dennerstein L, Hopper JL (2000) A prospective longitudinal study of serum testosterone, dehydroepiandrosterone sulfate, and sex hormone-binding globulin levels through the menopause transition^1^. J Clin Endocrinol Metab 85:2832–2838. 10.1210/jcem.85.8.6740 10946891 10.1210/jcem.85.8.6740

[43] Jones G, Barker A (2008) Reference intervals. Clin Biochem Rev 29(Suppl 1):S93-97 18852866 PMC2556592

[44] Grimstad F, Le M, Zganjar A, Flores D, Gourley E, May D et al (2018) An evaluation of reported follicle-stimulating hormone, luteinizing hormone, estradiol, and prolactin reference ranges in the United States. Urology 120:114–119. 10.1016/j.urology.2018.07.024 30056193 10.1016/j.urology.2018.07.024

[45] Jones GRD, Haeckel R, Loh TP, Sikaris K, Streichert T, Katayev A et al (2018) Indirect methods for reference interval determination – review and recommendations. Clin Chem Lab Med (CCLM) 57:20–29. 10.1515/cclm-2018-0073 29672266 10.1515/cclm-2018-0073

[46] Escobar-Morreale HF, Carmina E, Dewailly D, Gambineri A, Kelestimur F, Moghetti P et al (2012) Epidemiology, diagnosis and management of hirsutism: a consensus statement by the Androgen Excess and Polycystic Ovary Syndrome Society. Hum Reprod Update 18:146–170. 10.1093/humupd/dmr042 22064667 10.1093/humupd/dmr042

[47] Clinical and Laboratory Standards Institute, editor (2010) Defining, establishing and verifying reference intervals in the clinical laboratory: approved guideline. 3. ed. Wayne, Pa: Clinical and Laboratory Standards Institute

[48] Woolley T, Rutter E, Staudenmaier M (2023) Comparability and stability of serum creatinine concentration in capillary and venous blood. Br J Biomed Sci 80:11402. 10.3389/bjbs.2023.11402 37753533 10.3389/bjbs.2023.11402 PMC10519179

[49] Sodi R, Young N, Tetucci A, Woolley T, Shah V, Maund I (2025) Total prostate-specific antigen (PSA) testing in capillary samples: proof-of-principle and feasibility study for home self-testing for prostate cancer. J Appl Lab Med 10:250–258. 10.1093/jalm/jfae144 39723675 10.1093/jalm/jfae144

[50] Lutz SZ, Wagner R, Fritsche L, Peter A, Rettig I, Willmann C et al (2019) Sex-specific associations of testosterone with metabolic traits. Front Endocrinol 10:90. 10.3389/fendo.2019.00090 10.3389/fendo.2019.00090 PMC6425082 30930846

[51] Allen DB. Hormonal eligibility criteria for ‘Includes Females’ competition: a practical but problematic solution. Horm Res Paediatr. 2016;85:278–82. 10.1159/000444054 10.1159/000444054 26872015

[52] Sönksen PH, Holt RIG, Böhning W, Guha N, Cowan DA, Bartlett C, et al. Why do endocrine profiles in elite athletes differ between sports? Clin Diabetes Endocrinol. 2018;4:3. 10.1186/s40842-017-0050-3 10.1186/s40842-017-0050-3 PMC5804043 29445518

