# Supplementary material for: Redefining testosterone reference ranges for adult females
Source: J Endocrinol Invest. 2026 Jun 24;49(9):2339–49. doi: 10.1007/s40618-026-02929-w (PMC13498515; doi:10.1007/s40618-026-02929-w)
Supplement: Supplementary file 1 [file 40618_2026_2929_MOESM1_ESM.docx]

**Figure SI1:** Summary of the co–occurance of exclusion criteria represented with an UpSet plot. Set sizes (grey bars) are the total sample sizes excluded for each of the ten exclusion criteria. Red bars show the number of exclusions that do not co-occur. Blue shows all intersections of exactly two criteria, green shows intersections of exactly three criteria etc. For example, in total, 13,693 samples were excluded due to a diagnosis of polycystic ovarian syndrome. Of these, 4,727 samples met no other exclusion criteria, 2,252 co-occured with the BMI exclusion criterion, 1,065 co-occured with the BMI exclusion criterion AND the cycle length criterion etc. Plot generated using the function *upset* from the R package *UpSetR (*[*https://cran.r-project.org/package=UpSetR*](https://cran.r-project.org/package=UpSetR)*)*.
